# Discovery and quantification of anaerobic nitrogen metabolisms among oxygenated tropical Cuban stony corals

**DOI:** 10.1038/s41396-020-00845-2

**Published:** 2020-12-20

**Authors:** Andrew R. Babbin, Tyler Tamasi, Diana Dumit, Laura Weber, María Victoria Iglesias Rodríguez, Sarah L. Schwartz, Maickel Armenteros, Scott D. Wankel, Amy Apprill

**Affiliations:** 1 Department of Earth, Atmospheric & Planetary Sciences, Massachusetts Institute of Technology, Cambridge, MA 02139 USA; 2 Department of Marine Chemistry and Geochemistry, Woods Hole Oceanographic Institution, Woods Hole, MA 02543 USA; 3 Instituto de Ciencias del Mar de Cuba, Calle Loma No. 14 e/ 35 y 37, Alturas del Vedado, Plaza de la Revolución, La Habana, 10600 Cuba; 4 Program in Microbiology, Massachusetts Institute of Technology, Cambridge, MA 02139 USA; 5 Universidad Nacional Autónoma de México, Instituto de Ciencias del Mar y Limnología, Ciudad de México, CDMX 04510 México; 6 Universidad de La Habana, Centro de Investigaciones Marinas, 16 # 114, Playa, La Habana, 11300 Cuba

**Keywords:** Biogeochemistry, Biogeochemistry

## Abstract

Coral reef health depends on an intricate relationship among the coral animal, photosynthetic algae, and a complex microbial community. The holobiont can impact the nutrient balance of their hosts amid an otherwise oligotrophic environment, including by cycling physiologically important nitrogen compounds. Here we use ^15^N-tracer experiments to produce the first simultaneous measurements of ammonium oxidation, nitrate reduction, and nitrous oxide (N_2_O) production among five iconic species of reef-building corals (*Acropora palmata*, *Diploria labyrinthiformis*, *Orbicella faveolata*, *Porites astreoides*, and *Porites porites*) in the highly protected Jardines de la Reina reefs of Cuba. Nitrate reduction is present in most species, but ammonium oxidation is low potentially due to photoinhibition and assimilatory competition. Coral-associated rates of N_2_O production indicate a widespread potential for denitrification, especially among *D. labyrinthiformis*, at rates of ~1 nmol cm^−2^ d^−1^. In contrast, *A. palmata* displays minimal active nitrogen metabolism. Enhanced rates of nitrate reduction and N_2_O production are observed coincident with dark net respiration periods. Genomes of bacterial cultures isolated from multiple coral species confirm that microorganisms with the ability to respire nitrate anaerobically to either dinitrogen gas or ammonium exist within the holobiont. This confirmation of anaerobic nitrogen metabolisms by coral-associated microorganisms sheds new light on coral and reef productivity.

## Introduction

Corals reefs are critical environments hosting diverse marine life despite a confined footprint within Earth’s oceanic extent [1]. They thrive in shallow waters within a broadband from 30° S to 30° N of warm, sunlit, and relatively oligotrophic ocean waters [2]. Alarmingly, the success of coral reefs has floundered in recent decades in the face of increasing ocean temperatures, acidity, pathogens, and pressures from overfishing. These, along with other regional factors like anthropogenic nutrient runoff [3–5], have led to the degradation of 33–50% of the world’s reefs, placing a quarter of all marine species at risk for extinction [6]. The health of the coral-algal partnership depends on a complex microbial community of bacterial, archaeal, fungal, and viral associates, collectively termed the coral holobiont [7]. This holobiont maintains a delicate nutrient balance in order to support life among a reefs’ otherwise generally oligotrophic surroundings [8, 9]. One such nutrient, fixed nitrogen, is essential for the production of amino and nucleic acids and is a principal limiter of ocean productivity.

Tropical reef-building corals flourish despite the apparent constraints of their nutrient-poor environments [10, 11]. Indeed, the standing concentration of fixed “bio-available” nitrogen within these regions (mostly as nitrate) tends to be <1 µmol L^−1^ (Supplementary Fig. 1). These organisms then are accustomed to life at the precipice of resource availability and are aided by diazotrophic microbial associates that can continually supply new nitrogen to the ecosystem [12–14]. The marine nitrogen cycle comprises a finely tuned set of chemical transformations executed by a diverse community of diazotrophic, nitrifying, and denitrifying microorganisms. Even though functional gene assays have identified these microbes in reef-building corals [15], direct evidence of their function within the overall ecosystem has not been fully explored. The nitrogen-fixing microbes responsible for the conversion of otherwise inert N_2_ to readily available ammonium (NH_4_^+^), may allow corals to thrive despite low standing stocks of fixed nitrogen [16]; however, the roles of other nitrogen cycling pathways remain almost entirely unknown [17]. Given the importance of nitrogen to cell growth and its scarcity on reefs, the coral-based nitrogen cycle is likely to include the suite of processes observed in the global oceans and in other marine invertebrates [18].

**Fig. 1:**
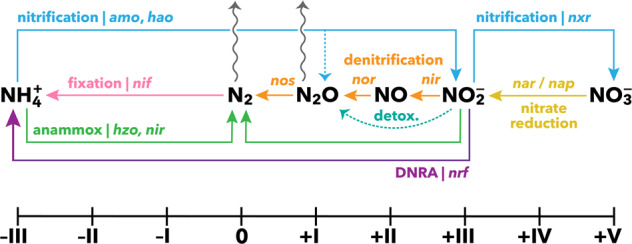
Potential nitrogen cycle among coral organisms. The multiple inorganic nitrogen compounds are plotted according to the oxidation state of the nitrogen. The biologically mediated pathways are denoted by the functional gene responsible for each dissimilatory conversion. Notably, there are three major avenues for N_2_O production, all of which can lead to a flux from the surface ocean: nitrification (through decomposition of a hydroxylamine intermediate or via nitrifier denitrification), denitrification, and non-energy-yielding detoxification of nitrite.

Nitrogen is a globally important nutrient and proximal limit on marine photosynthesis across much of the oceans [19, 20]. The overall budget of fixed nitrogen in the oceans is predominantly set by two main microbial pathways: nitrogen fixation as a source and denitrification as a sink [21]. Nitrogen fixation in the surface oceans, including tropical reefs, is largely attributed to autotrophic cyanobacteria [22]. Meanwhile, fixed nitrogen loss, has been generally considered limited to anoxic systems [23], including coral reef sediments [24]. A main contributor to this nitrogen loss is canonical denitrification, the sequential reductions of nitrate (NO_3_^−^) to nitrite (NO_2_^–^) to nitric oxide (NO) to nitrous oxide (N_2_O) and finally to dinitrogen gas (N_2_). The steps of denitrification are catalyzed by the enzymes nitrate reductase, nitrite reductase, nitric oxide reductase, and nitrous oxide reductase, encoded by the genes *nar*, *nir*, *nor*, and *nos*, respectively [25]. The enzymes that catalyze these nitrogen transformations display varying sensitivities towards oxygen [26], and so their activity can be dependent on local O_2_ microenvironments. Another energetically favorable nitrate reduction pathway, dissimilatory nitrate reduction to ammonium (DNRA), is encoded by the *nrf* gene and is an anaerobic process that has been detected across diverse marine environments [27–29]. This dynamic cycle comprises many pathways distributed among different organisms, exchanging inorganic nitrogen metabolites through their dissimilatory metabolism (Fig. 1).

Broadly, the nitrogen cycle includes both aerobic and anaerobic pathways, with denitrification requiring low O_2_ [30, 31], N_2_ fixation poisoned by the presence of O_2_ [32], but nitrification requiring O_2_ [33]. As a result, these processes are separated in space and time due to the inhibition of enzymes like nitrate reductase, the catalyst for the first step in denitrification, by oxygen [34, 35]. The diurnal variations in oxygen concentrations observed within the mucus films and tissues of corals may permit suboxic metabolisms in otherwise well-oxygenated bulk systems. Coral reefs exhibit dramatic diurnal fluctuations in dissolved oxygen [36], especially at the sub-millimeter scale within the gastric cavities and boundary tissue layers of individual corals, which can oscillate between full saturation in daylight to anoxia at night even as ambient water remains constant [37–39]. Indeed, mucus can act as an organic carbon source enabling rapid respiration [40] and as an oxygen retardation boundary to create localized anoxia [41]. Even though these low oxygen conditions prime reefs for nitrate reduction and denitrification, the ability of tropical reef microbial symbionts to conduct these processes has received less attention than reef carbonate sediments [24], oyster reefs [42], and tropical sponges [43].

There have been few direct rate measurements of fixed N loss despite the identification of microbial communities associated with nitrate reduction [13, 15, 44]. While suggestive, the molecular evidence highlights a need for direct rate measurements of denitrification in and isolation of coral individuals from tropical reefs in order to confirm the activity of suboxic metabolic pathways. Analogously, cold-water corals have been shown to exhibit a nitrogen cycle comprising fixation, nitrification, and denitrification [45–47]. Yet, these cold-water corals survive in deeper, darker, colder, and more nutrient-rich waters and are usually devoid of algal symbionts [48], making extrapolation of those results to the tropical surface tenuous. Tropical reef sponges harbor a markedly complex and tightly interwoven microbially-driven nitrogen cycle [43, 49], but sponges can actively modulate their internal oxygen concentrations to support both aerobic and anaerobic microbes [50]. In oligotrophic tropical reef environments, which neither conserve nor export significant quantities of inorganic nutrients [51], an active nitrogen cycle is vital for maintaining a highly productive system. The presence of denitrifying pathways in analogous organisms and characteristic oxygen fluctuations that can prime micro-zones for denitrification suggest a promising capacity of tropical corals to host anaerobic nitrogen metabolisms.

Here, in the well-preserved coral reef system Jardines de La Reina (Cuba) that generally harbors low standing stocks of dissolved inorganic nitrogen [52], we investigate the in-situ cycling rates of inorganic nitrogen in five important Caribbean coral species using a multitracer experimental approach. We directly measure rates of nitrate reduction, ammonium oxidation, and nitrous oxide production from each possible inorganic nitrogen source via ^15^N tracer amendments. We hypothesize that nitrogen cycling variability is related to coral species. In addition, we isolated anaerobic nitrate-consuming bacteria in order to more fully understand the roles that denitrifying organisms have in governing coral reef dynamics and health.

## Materials and methods

### Study site

**Fig. 2:**
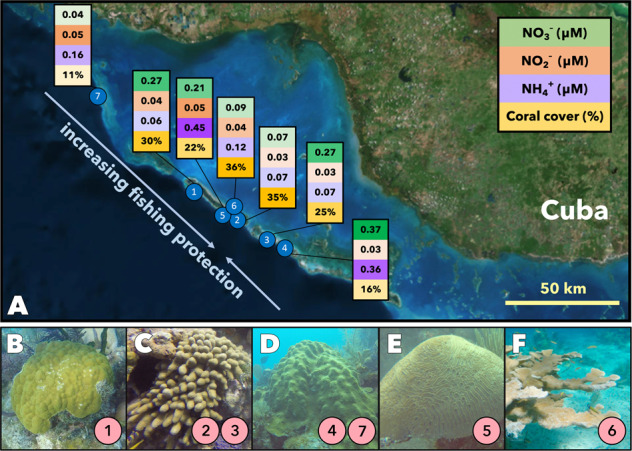
Coral and reef water sampling in the Gardens of the Queen. **A** Locations of the seven sites that corals were sampled from for the incubation experiments. Shown are the measured inorganic nitrogen concentrations as well as the quantified coral coverage for the given section of reef, with higher and lower concentrations (or coverage) represented by the dark and light colors, respectively. Coral species sampled at each site (indicated in the pink encircled number): **B** *Porites astreoides*, **C** *Porites porites*, **D** *Orbicella faveolata*, **E** *Diploria labyrinthiformis*, and **F** *Acropora palmata*.

The Jardines de la Reina (Gardens of the Queen) is a relatively pristine Caribbean reef system located in the southeastern coast of Cuba, sheltered historically due to its remote nature from the Cuban coastline and protected officially since 1996 by the Cuban government. It hosts lower degrees of local pollution, fishing pressures, and other anthropogenic influences than are present in other areas of the Caribbean [53]. Many reef ecosystems elsewhere in the region, such as Jamaica and the Florida Keys, have been heavily impacted by these human influences and subsequent coral disease outbreaks [54, 55]. The Jardines de la Reina stands in stark contrast, as an extensive National Park (a category of marine protected area) that has resulted in a well-preserved ecosystem. Additionally, the Jardines de la Reina maintains endangered coral species, like *Acropora palmata*, that have experienced precipitous population declines elsewhere in the Caribbean [56]. Fish and coral densities are highest in the central regions of the Jardines de la Reina and slightly lower at the northwestern- and southeastern-most parts, where fishing is permitted on a limited albeit regulated basis [57]. Sampling was conducted aboard the M/V *Alucia* in November 2017. Seven sites were chosen for sampling from a variety of reefs, and due to cruise and experimental logistical constraints, one coral species was chosen per site based on prevalence and availability (Fig. 2). All of the sites sampled were forereefs (9–14 m), except *A. palmata* which was collected from a shallower 3 m reef.

### Reef metadata collection

Samples for NO_x_^–^ (as nitrate + nitrite), nitrite, and ammonium concentrations at each of the sites were collected at reef depth into clean 30 mL HDPE bottles and frozen at −80 °C until analysis (within 2 months) using standard colorimetric methods by Oregon State University [58, 59]. At each site, scuba divers conducted benthic surveys to estimate the percent coral cover relative to other reef substrates, such as macroalgae, sponges, rocks, and sand. The composition of each substrate (in cm) was estimated along a transect tape at 1 m intervals for a total distance of 10 m, and replicated 8–10 times for an individual reef site.

### Coral ^15^N incubation experiments

Coral fragments for incubation were collected from a single colonial head per site by hammer and chisel. These fragments were maintained in WhirlPak bags and returned to the surface where they were kept at seawater temperature until introduction to the incubation vessels. Coral pieces were largely clean and lacking epiphytic and endolithic algae or sponges, confirmed by visual inspection after the experiments. Pieces ranged between 1 and 10 cm^3^ in volume, depending on the species sampled, and generally did not vary in size within an incubation by more than a factor of four. The corals sampled were: *Porites astreoides* (site 1), *Porites porites* (sites 2 and 3), *Orbicella faveolata* (sites 4 and 7), *Diploria labyrinthiformis* (site 5), and *Acropora palmata* (site 6).

The corals were transferred into sterile on-deck incubators within two hours of removal from the reef. Each vessel was a 12 cm × 10 cm × 3 cm (360 mL) rigid, chemically inert, polycarbonate container selected for its relative clarity across the UV and visible light spectrum (Pelican Products, Torrance, CA) (Supplementary Fig. 2). This size chamber was selected to maximize the ability to detect ^15^N transformations happening in/on the coral fragment. The containers were modified to include a bulkhead compression port fitted with a gray chlorobutyl rubber septum and a Viton rubber gasket. The port allowed each container to be filled completely without residual air bubbles and also provided a means of injecting ^15^N tracers. Each customized container passed a 24-hour quality control test by not permitting the exchange of dye-injected water inside the container with bulk water outside. For each incubation, at least one coral piece was placed in a container that was then filled completely with reef water collected directly from the reef where the parent coral colony had been situated using a 10 L Niskin bottle. Using ambient reef water ensured that the chamber contained a similar chemical, microbiological, and metabolomic profile to the corals’ natural environment and thus was more reflective of on-reef conditions. These chambers, filled with reef seawater and corals, were incubated at approximate reef temperatures maintained by supplying continuous flowing surface seawater to aquaria on the research vessel. In all, the time from the collection of corals on the reef to the start of the incubation via inoculation was approximately two to three hours.

After each container was sealed, each was amended to a final concentration of 1 μmol L^−1^ of one of three tracer species: K^15^NO_3_, Na^15^NO_2_, or ^15^NH_4_Cl (>99%, Cambridge Isotope Laboratories, Tewksbury, MA). In addition, natural abundance additions of the complementary dissolved inorganic nitrogen (DIN) compounds were amended to serve as carriers and to standardize inoculation amendments across experiments. As such, 1 mmol L^−1^ stock solutions of the appropriate ^14/15^N compounds were mixed to make three combined tracer solutions: each contained one of three ^15^N labels and the two ^14^N carriers. Final concentrations were attained by a single injection of 1 mL of the combined tracer solutions into the incubation vessels via a 22 G needle through the septum. The ^15^NO_3_^–^ tracer assessed rates of reduction to NO_2_^–^, N_2_O, and N_2_ (denitrification), the ^15^NO_2_^–^ tracer tracked the rate of its reduction to N_2_O and N_2_, and the ^15^NH_4_^+^ tracer indicated the rates of oxidation to NO_2_^–^ (nitrification), N_2_O (nitrifier denitrification) and N_2_ (anammox). For each set of experiments, six incubations were performed per ^15^N label for a total of 18 individual incubations per site. Twelve of these contained corals from the designated site (four per ^15^N tracer), and an additional six incubations were seawater-only controls to measure ambient DIN cycling rates in reef water alone for comparison.

Incubations were separated into one of two different light treatments in order to investigate the impact of irradiance on DIN cycling rates. Half of the incubations (three per tracer) were covered with dark mesh to reduce incident light levels at the surface and better approximate ambient illumination at the coral’s natural depth—this is referred to as the “ambient light” treatment—while the other was covered with opaque, black plastic in order to estimate the effect of lack of photosynthesis on the coral’s biogeochemical nitrogen cycling profile, *i.e*., the “dark” treatment. Corals in the “ambient light” treatment were otherwise subject to the normal diurnal cycle. Light levels were confirmed using a photosynthetically active radiation sensor (Vernier, Beaverton, OR) and indicated that the mesh used in our “ambient light” treatment attenuated incident light by 80%, thus approximating the light levels that corals would receive at a depth of 8–10 m [60, 61].

At each site, seawater samples were also collected to serve as initial timepoints; one was collected for each ^15^N tracer experiment. Each was amended to a final concentration of 1 μmol L^−1 15^NO_3_^**–**^, ^15^NO_2_^**–**^ or ^15^NH_4_^+^ and 1 μmol L^−1^ of the complementary ^14^N species with the same isotopic tracer mixture injected into the experiments. This inoculated reef water was then immediately split between each of two sample bottles: a 30 mL acid-washed high-density polyethylene (HDPE) bottle and a 30 mL glass serum bottle, both of which had been pre-aliquoted with 50 μL of 50% (by weight) ZnCl_2_ as a preservative. 25 mL was injected into each bottle and the glass serum bottle was then crimped closed. This gastight sample was used for the isotopic measurement of dissolved N_2_O and N_2_, whereas the sample preserved in the HDPE bottle was used for the measurement of dissolved NO_3_^–^ and NO_2_^–^.

Corals were incubated for a 24-hour period so as to capture a full diurnal cycle as well as to allow enough time for processing of the nitrogen inocula, but also provide a short enough window to minimize bottle effects typically associated with lengthy incubations. At the end of each incubation, the bulkhead cap was unscrewed and water samples were collected from each vessel. A total of 50 mL was split between the HDPE and glass serum bottles as in the collection of the initial timepoint samples. Once water samples had been taken, corals were removed from the vessels, wrapped in aluminum foil, and frozen at −80 °C until processing.

### Coral fragment surface areas and other physical attributes

Estimated dry tissue weights, skeletal volumes, polyp counts, and active surface areas were determined for each coral sample. First, each coral piece was airbrushed (Paasche Model H, Chicago, IL) at 80 to 100 psi with 0.2 μm filter sterilized phosphate-buffered saline to remove coral mucus and tissue. The slurry was vortexed for 20 min to homogenize and a fraction of it was then dried and weighed. Cleaned coral skeletons were measured for surface area, skeletal volume, and polyp counts, all of which were more reliable than the dry weight estimates (Supplementary Table 1). Coral active surface areas were estimated by wrapping each cleaned coral skeleton in a monolayer of aluminum foil and weighing the resultant mold [62]. Foil was used to cover only the area of coral that would have had live tissue, and all measurements used a single roll of standardized foil. Although widely adopted, this protocol does not capture finer details of structure inside of polyps and on species with particularly complex polyp morphologies, like *O. faveolata* [63]. Polyp density was determined by individually counting every polyp on each coral piece via visual inspection and then normalizing by the surface area estimates. Coral volumes were measured by water displacement in 25 or 50 mL graduated cylinders, depending on coral fragment size.

### ^15^N mass spectrometry

The isotopic compositions of dissolved nitrogen species, ^15^N/^14^N, were determined using a continuous flow IsoPrime 100 isotope ratio mass spectrometer (IRMS; Elementar Americas, Inc., NY). Ammonium oxidation to nitrite and nitrate reduction to nitrite samples were measured after quantitative conversion of sample nitrite to N_2_O in a buffered sodium azide solution [64, 65]. A 1:1 mixture of 2 mol L^−1^ sodium azide and 20% glacial acetic acid was added to each sample in a gastight and crimp-sealed 20 mL glass vial (Restek Corp., Bellefonte, PA). Each sample received 10% azide reagent by volume, and was allowed to react for 1 h at room temperature. Samples were then neutralized with 0.1 mL of 6 mol L^−1^ sodium hydroxide per milliliter of sample before introduction into the IRMS. An internally calibrated nitrite standard (−1.7‰) was analyzed for every seven samples to account for instrument drift. Due to the low DIN concentrations in our samples, a known amount (~10 nmol) of NO_2_^–^ was added as a ‘carrier’ during processing in order to increase the final amount of N_2_O in each sample to least 10 nmol and increase signal size and improve measurement fidelity. The isotopic δ^15^N composition of this carrier was well characterized (−1.7‰ for the internal sodium nitrite standard), and all carrier additions were made gravimetrically to minimize errors. The analytical precision for δ^15^N measurements using the denitrifier and azide methods are typically 0.3‰ or better.

Potential N_2_O and N_2_ production rates from each incubation were measured directly from the gastight serum bottle samples. N_2_ measurements were performed first by drawing 1 mL of the headspace into a Hamilton gastight syringe and injecting directly into an injection port on the IRMS prep system, which included chromatic separation of O_2_ and Ar from N_2_ (Mol Sieve 5 Å, 1/8” OD x 2 m). Due to the substantial headspace of air in the serum bottles during filling, the detection limit of N_2_ production was 1 μmol L^−1^ d^−1^ (40–200 nmol cm^−2^ d^−1^). The same serum bottle was then analyzed for ^15^N–N_2_O production by sparging with He at a rate of 100 mL min^−1^ for 15 min to quantitatively strip any N_2_O out of the sample. Each sample was also purged inline with a second vial (“daisy-chained”) containing N_2_O produced by the sodium azide method from the internal nitrite standard to accumulate sufficient N_2_O to resolve on the IRMS. The liberated N_2_O was concentrated in a liquid nitrogen cryotrap before introduction to the IRMS. The detection limit for N_2_O production was 10 nmol L^−1^ d^−1^ for ^15^NO_2_^–^ and ^15^NO_3_^–^ additions and 2 nmol L^−1^ d^−1^ for ^15^NH_4_^+^ additions.

Potential rates were calculated based on the difference between the initial and final timepoint measurements. Coral surface areas were used to normalize initial rate measurements from each incubation to a rate per surface area of coral tissue:

1 $${\mathrm{R}}^\prime = {\mathrm{V}} \times\, \frac{{{\mathrm{R}}_{\mathrm{C}} - {\mathrm{R}}_{{\mathrm{SW}}}}}{{{\mathrm{SA}}}},$$

where *R*′ is the surface area normalized transformation rate attributable to coral-associated nitrogen metabolism. Normalization by surface area (*SA*) is important because it most clearly accounts for the quantity of microbial habitats within coral mucus and tissue. As all corals were incubated in reef seawater of volume *V*, potential rates from coral incubations (*R*_*C*_) were assumed to be a combination of both coral and seawater transformation rates (*R*_*SW*_). Thus, the rates from seawater-only incubations (generally an order of magnitude lower than the coral-associated rates) were subtracted in order to isolate the nitrogen cycling potential of direct coral associates. The volume was determined as the size of the incubation vessel correcting for the displacement by the coral fragment(s), but the coral volume accounted for generally ≲1% of the total chamber.

### Isolation and identification of anaerobic nitrate reducers

Separate from the rate experiments, mucus and tissue samples from four healthy coral colonies*—Montastraea cavernosa*, *O. faveolata, P. astreoides*, and *D. labyrinthiformis*—were collected at site 4 (one of the sites sourced for the *O. faveolata* incubations), and inoculated shipboard on marine broth agar plates amended with 500 µmol L^−1^ nitrate. Inoculated plates were incubated in the dark at ~22 °C in a BD GasPak EZ container system (Becton, Dickinson and Company, Franklin Lakes, NJ) with five oxygen-scrubbing sachets. The anaerobic indicator showed that anoxic conditions were established after less than one day. Colonies were picked after approximately three weeks, re-plated, and regrown anaerobically on marine agar 2216 plates. Any plates with uniform colony growth were picked, transferred to liquid media for a final growth, and cryopreserved in 25% glycerol. Some plates with no growth after a week were stored at 4 °C. After a year, some preserved plates had visible growth. These colonies, all *Psychrobacter* spp., were isolated using the same processing protocols. Cryopreserved isolates in glycerol were re-streaked in 2216 media amended with 10 mmol L^−1^ nitrate and incubated at 37 °C in anaerobic conditions until colony growth was visible to confirm viability. Colonies were then picked and grown in liquid 2216 media anaerobically for DNA extraction. This process resulted in the growth of 29 isolation targets.

### Phenotypic assay for nitrogen oxide reduction ability

A phenotypic assay was developed to verify the capability of the isolates to reduce nitrate and/or nitrite. Deep 96-well plates were flushed for 15 minutes with nitrogen gas in a BD GasPak EZ container system and allowed to degas with oxygen-scrubbing sachets for two days. After degassing of the plastic plates, 1.880 mL of N_2_-equilibrated marine broth 2216 liquid media was added to each well. Preserved isolates were revived 24 hours prior to the experiments by inoculating in anoxic liquid media. Assays with nitrate or nitrite were conducted, with each well receiving a final concentration of one of the compounds of 50 µmol L^−1^. Subsequently, 20 µL of previously revived isolate stock was amended to triplicate wells. Multiple plates were constructed in order to take three sacrificial timepoints for nitrite and four sacrificial timepoints for nitrate at different times. After inoculation, plates were stored in individual plastic bags with oxygen-scrubbing catalysts and incubated at 30 °C under anaerobic conditions. Nitrite was measured on sacrificed plates via the Griess reaction [58] to visually determine if nitrate was consumed to produce nitrite and if nitrite was completely metabolized. For treatments with nitrate, the timepoints were: 4, 8, 24, and 48 hours after inoculation; for nitrite treatments, the timepoints were: 24, 48, and 144 hours after inoculation.

### DNA extraction and 16S rRNA identification

Bacterial isolates were grown aerobically in 50 mL of marine broth 2216 liquid culture overnight and centrifuged (10,000 × g) for 10 minutes. Afterwards, DNA from the cell pellet was extracted following the DNeasy Powerlyzer Microbial Kit protocol (Qiagen, Hilden, Germany). DNA quantity and purity were determined with a NanoDrop UV/vis spectrophotometer (NanoDrop Technologies, Wilmington, DE). The small subunit ribosomal RNA gene was amplified with Quick-Load Taq 2X Master Mix (New England BioLabs, Ipswich, MA) using universal bacterial 8 F/1492 R primers and sent to Eton Bioscience for Sanger DNA sequencing using primers 8 F and 907 R. From the combined 16S sequencing and phenotypic assay results, the isolation targets were culled to 15 potential unique organisms for whole genome sequencing.

### Whole genome sequencing, genome assembly, and annotation

Isolate DNA was purified and submitted to the BioMicro Center at the Massachusetts Institute of Technology for Whole Genome Sequencing (WGS). Library preparation for Illumina sequencing was done using the ligation based NEBNext Ultra II kit (New England BioLabs, Ipswich, MA). Samples were sequenced using paired-end MiSeq sequencing (V3 kit providing 300 bp forward and 300 bp reverse reads) (Illumina Inc., San Diego, CA). Genome assembly was accomplished by using the following pipeline: Trimmomatic [66] was used to clip ends of the Illumina adapters from the raw reads with the following parameter set: Illuminaclip: TruSeq3-PE-2.fa:2:20:10, Leading: 3, Trailing: 3, SlidingWindow: 10:20, Minlen: 36. Bayeshammer (part of the SPAdes package) [67] was used for error correcting and clustering. After error-correction of low-quality reads, a rough assembly and annotation was completed using MG-RAST [68] to compare with the identities obtained previously from 16S rRNA gene amplification. MegaHit [69] was used to assemble the genome, MaxBin [70] binned the assembled genomes and assigned operational taxonomic units (OTUs), and CheckM [71] was used to assesses genome quality, estimate completeness and provide genetic identification from marker genes (these are the reported identities). Annotation of the assembled genomes was done with the program PROKKA [72]. Genomes are available from the NCBI Genbank database, accession number PRJNA646503.

Organisms were characterized by the presence or absence of identified nitrate reductase (*nar* and *nap*), denitrifier nitrite, nitric oxide, and nitrous oxide reductases (*nir*, *nor*, and *nos*, respectively), and dissimilatory nitrite reductase to ammonium (*nrf*) genes. In short, genetic capacity was identified using HMMR v. 3.3 [73]. Hidden Markov Models (HMM) for *narG*, *napA*, *nirS*, *nirK*, *norB*, *norC*, *nosZ*, and *nrfB* were obtained from the TIGRFAMs resource maintained by the J. Craig Venter Institute.

## Results

### Shipboard ammonium oxidation and nitrate reduction rates

**Fig. 3:**
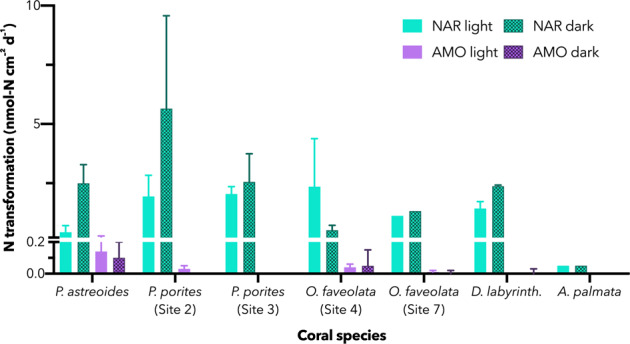
Coral-associated rates of nitrogen metabolism. Duplicate rates of nitrate reduction (green, NAR) and ammonium oxidation (purple, AMO) were detected in association with multiple coral species (*n* = 2 per experiment and light treatment). These rates tended to be higher when incubated in full darkness (hashed bars) compared to diurnally varying light conditions (solid bars). Rates of ammonium oxidation tended to be an order of magnitude slower than nitrate oxidation. Coral-associated rates were determined by subtracting the rates from seawater controls (*n* = 2 per light treatment). Error bars denote the range of measurements.

Inorganic nitrogen concentrations were low throughout the sites, with ammonium and nitrate each <0.5 µmol L^−1^ and nitrite ≤0.06 µmol L^−1^ (Fig. 2). Coral cover at the sampling sites ranged from 11 to 35% (Fig. 2). At each site, the chosen species was sampled according to availability and abundance to minimize impact of destructive sampling. Overall, coral cover was higher at these sites (25% on average) as compared with much of the Caribbean (currently estimated at 10–16%) [52, 74]. Separate shipboard nitrogen tracer experiments with *P. astreoides*, *P. porites*, *D. labyrinthiformis*, *A. palmata*, and *O. faveolata* showed that coral-associated nitrate reduction to nitrite were detected in all coral species, and ammonium oxidation was detected in all species except *A. palmata* (Fig. 3, Supplementary Table 2). Coral-associated potential rates were calculated by subtracting the rate from the seawater-only incubation and normalizing to the surface area of the coral fragment incubated. The seawater rates (no coral fragment) were generally an order of magnitude slower than the coral-associated rates. Measured coral-associated rates of nitrate reduction, a suboxic reductive process, (generally > 1 nmol of N cm^−2^ d^−1^) were greatly in excess of ammonium oxidation, an oxidative process (<0.2 nmol of N cm^−2^ d^−1^). Moreover, whereas nitrate reduction rates were concentrated within the coral-amended incubations (*t*-test, *p* = 0.002), the ammonium oxidation rates were indistinguishable between coral-amended and seawater-only incubations (*t*-test, *p* = 0.87). Indeed, across all species sampled, ammonium oxidation rates displayed no coherent trend with either underlying nutrient concentrations, reef health as determined by coral coverage, host species, or light treatment.

Potential nitrate reduction rates were much faster than those of ammonium oxidation, changing the total seawater within an incubation chamber by up to 150 nmol L^−1^ d^−1^. The seawater-only (no coral) nitrate reduction rates accounted for only 2.8 ± 1.0% (standard error, se) of the observed coral-associated rates. Thus, the vast majority of this high nitrate reduction rate was associated with the corals directly rather than by planktonic organisms subsisting in the bulk seawater. Potential nitrate reduction rates were further consistent across all species sampled with the exception of *A. palmata*, where nitrate reduction rates were measured to be just 0.05 nmol cm^−2^ d^−1^. Nitrate reduction rates further show a dependence on light levels, with the dark incubations fostering higher rates than those undergoing natural diel light conditions. Pointedly, the dark incubations showed 2.00 ± 0.52 (se) fold higher nitrate reduction rates than their diurnal counterparts.

### Nitrous oxide production rates from multiple tracers

We were unable to quantify denitrification and anammox rates to dinitrogen gas due to experimental limitations (^29^N_2_ production below detection, not shown), but the potential rates of these processes could be constrained to <40–200 nmol cm^−2^ d^−1^ based on the range of surface areas, or less than 1 μmol L^−1^ d^−1^ within the water. This does not mean that these metabolisms were absent, as the other transformation rates (including N_2_O production) were well below this threshold (20–70 nmol L^−1^ d^−1^ for nitrate reduction and 10–80 nmol L^−1^ d^−1^ for N_2_O production). By not purging the ample N_2_-background from our incubation vessels [75], our experimental design did not allow for the detection of ^15^N–N_2_ production.

**Fig. 4:**
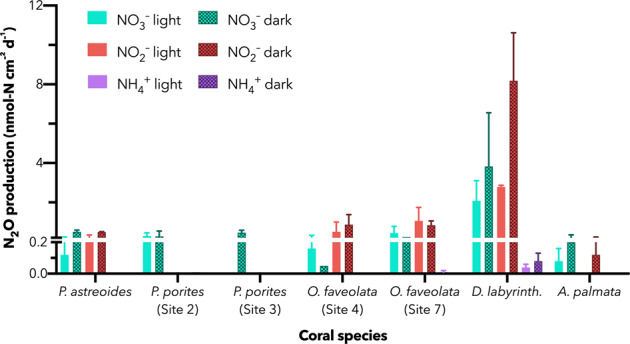
Rates of N_2_O production from multiple metabolic routes. Duplicate N_2_O production rates are determined from nitrate (green), nitrite (red), and ammonium (purple) sources. The rates of production via reductive denitrifying processes, i.e., nitrate and nitrite reduction far exceed the rates from ammonium-based nitrifier denitrification. Error bars denote the range of measurements. *D. labyrinthiformis* harbored the fastest rates of denitrification, followed by *O. faveolata*. The *Porites* spp. and *A. palmata* indicated low but detectable rates on order of 0.2 nmol cm^−2^ d^−1^. Production of N_2_O from ammonium was only observed for *D. labyrinthiformis*, and at rates 20 and 40 times slower than for nitrate and nitrite reduction, respectively. Dark incubations (hashed bars) were generally faster than those under diurnally varying light (solid bars).

Production of ^15^N–N_2_O was broadly detected from incubations inoculated with ^15^NO_3_^–^ and ^15^NO_2_^–^ (Fig. 4, Supplementary Table 3), but not generally from those inoculated with ^15^NH_4_^+^. The exception was *D. labyrinthiformis* which produced measurable but small amounts of N_2_O from ammonium. When detected, *Porites* spp. generated N_2_O from NO_3_^–^ and NO_2_^–^ at rates averaging 0.37 ± 0.06 (se) nmol L^−1^ d^−1^ for the three incubations. Meanwhile, *O. faveolata* averaged 0.62 ± 0.15 nmol L^−1^ d^−1^ across its two incubations. Despite only sampling *D. labyrinthiformis* and *A. palmata* from one site each, their observed N_2_O production rates were consistent from NO_2_^–^ and NO_3_^–^ for each species, generating 4.2 ± 1.1 and 0.20 ± 0.06 nmol L^−1^ d^−1^ N_2_O, respectively. Due to the trace nature of N_2_O relative to N_2_ gas, the quantification of its production had a much lower limit of detection.

**Fig. 5:**
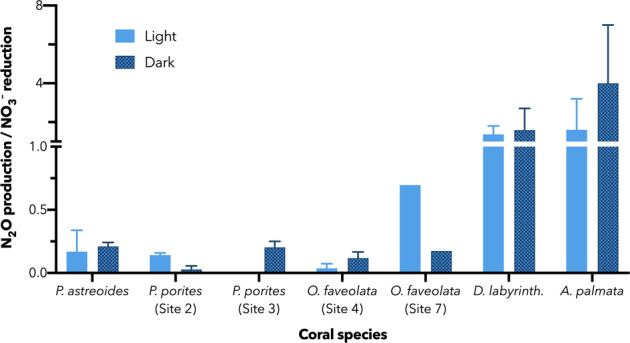
Ratios of N_2_O production to NO_3_^–^ reduction. A ratio of unity (1.0) indicates the equal reduction of nitrate to nitrite to N_2_O, implying coupled reactions. Within the margin of error, *D. labyrinthifo*rmis and *A. palmata* indicate this occurrence, although *D. labyrinthiformis* displays much faster rates than *A. palmata*. Ratios less than one imply likely recycling of nitrogen, either via uptake or nitrification. Error bars denote range of duplicate measurements except in the case of *O. faveolata* (Site 7), where one replicate was lost.

Strikingly, a similar enhancement of potential rates in the dark incubation as seen for NO_3_^–^ reduction was observed for N_2_O production via denitrifying metabolisms (NO_2_^–^ and NO_3_^–^-derived pathways). The dark incubation rates averaged 1.7 ± 0.4 (^15^NO_3_^–^ addition) and 2.0 ± 0.3 (^15^NO_2_^–^ addition) fold higher than their corresponding diurnally varying light experiments. This effect is especially pronounced for *D. labyrinthiformis* (Fig. 4). *D. labyrinthiformis* displays not only reproducible enhancement of N_2_O production from both NO_2_^–^ and NO_3_^–^ in the dark, these rates are within the margin of error of each other for both light and dark treatments (Fig. 5), indicating quantitative conversion of NO_3_^–^ to NO_2_^–^ to N_2_O.

### Isolation and phenotyping of anaerobic nitrate reducing coral symbionts

From the four coral species surveyed, we obtained 15 nitrate reducing bacterial isolates comprising five genus-level identities, all from the phylum Proteobacteria (Table 1). Of the organisms identified, *Marinobacter* spp. were found associated with *M. cavernosa*, *O. faveolata*, and *P. astreoides*, and in all cases were denitrifiers with the full complement of denitrification genes per our HMM searches. *Vibrio harveyi* was found in association with each coral species, and in all cases had the ability to reduce nitrate to ammonium (DNRA). *Pseudoalteromonas* spp., *Psychrobacter* spp., and *Vibrio* sp. were found as well and each isolate was able to reduce nitrate to nitrite. Yet of these species, only the bacteria associated with *P. astreoides* were able to reduce nitrite via the phenotypic assay. Of the organisms isolated, each was able to consume nitrate and generate nitrite, and only the *Pseudoalteromonas* sp. and *Psychrobacter* spp. associated with *D. labyrinthiformis* and *M. cavernosa* were negative for nitrite reduction ability (Supplementary Fig. 3). Of the isolates obtained, 80% contained membrane-bound nitrate reductase *narG*, 60% contained periplasmic nitrate reductase *napA*, and 43% had both. Moreover, the only occurrences of nitrite (*nirS* or *nirK*) and nitric oxide reductase (*norBC*) were found in association with each other, and only for *Marinobacter* spp. In most cases, the results from the phenotypic assay agreed with the genome annotations made through targeted HMM analyses.

**Table 1:** Identities and function of the multiple facultative nitrate reducing isolates obtained from the Gardens of the Queen corals.

Coral species	Bacterial identity^a^	Annotated genes	Annotated function	Phenotype	NCBI GenBank accession number
*Diploria labyrinthiformis*	*Pseudoalteromonas sp. A*	*narG, nosZ*	Nitrate reducer	Nitrate reducer	SAMN15545061
	*Psychrobacter sp. A*	*napA*	Nitrate reducer	Nitrate reducer	SAMN15545062
	*Psychrobacter sp. B*	*narG, napA*	Nitrate reducer	Nitrate reducer	SAMN15545064
	*Vibrio harveyi A*	*narG, napA, nrfB*	DNRA	Nitrate and nitrite reducer	SAMN15545055
	*Vibrio harveyi B*	*narG, napA, nrfB*	DNRA	Nitrate and nitrite reducer	SAMN15545056
*Montastraea cavernosa*	*Marinobacter sp. A*	*napA, nirS, norBC, nosZ*	Denitrifier to dinitrogen	Nitrate and nitrite reducer	SAMN15545053
	*Pseudoalteromonas sp. B*^b^	*—*	—	Nitrate reducer	SAMN15545054
	*Vibrio harveyi C*	*narG, napA, nrfB*	DNRA	Nitrate and nitrite reducer	SAMN15545059
*Orbicella faveolata*	*Marinobacter sp. B*	*narG, napA, nirS, norBC, nosZ*	Denitrifier to dinitrogen	Nitrate and nitrite reducer	SAMN15545058
	*Vibrio harveyi D*	*narG, napA, nrfB*	DNRA	Nitrate and nitrite reducer	SAMN15545057
	*Vibrio harveyi E*	*narG, napA, nrfB*	DNRA	Nitrate and nitrite reducer	SAMN15545063
*Porites astreoides*	*Marinobacter sp. C*	*napA, nirS, norBC, nosZ*	Denitrifier to dinitrogen	Nitrate and nitrite reducer	SAMN15545065
	*Pseudoalteromonas sp. C*	*narG*	Nitrate reducer	Nitrate and nitrite reducer	SAMN15545060
	*Vibrio sp*.	*narG, napA*	Nitrate reducer	Nitrate and nitrite reducer	SAMN15545051
	*Vibrio harveyi F*	*narG, napA, nrfB*	DNRA	Nitrate and nitrite reducer	SAMN15545052

The function is ascribed by the presence of *narG(napA)/nirS/norBC/nosZ* genes for denitrifiers, or *nrfB* for DNRA. Nitrate reducers both lacked nitrite reduction genes (*nir* or *nrf*) and did not reduce nitrite in the phenotypic tests.

^a^Letters indicate different isolated strains of the same genus- or species-level identity. Accession numbers are for NCBI GenBank, BioProject number PRJNA646503.

^b^This Pseudoalteromonas is negative for all examined dissimilatory nitrogen metabolism genes although phenotypically it acts as a nitrate (but not nitrite) reducer.

## Discussion

**Fig. 6:**
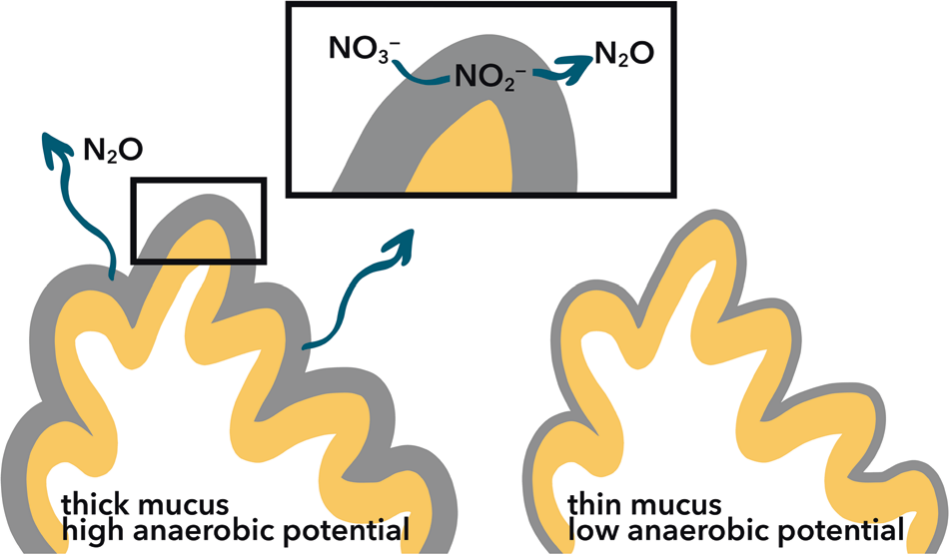
N_2_O production schematic within corals. Thick mucus reduces oxygen supply and enables faster respiration, resulting in high anaerobic potential of associated microbes. These microbes must then reduce nitrate to nitrite and N_2_O (or ammonium) for energy. Thin mucus does not enable the same anaerobic metabolic rates as oxygen is not as sufficiently reduced.

These experiments reveal new evidence for anaerobic nitrogen cycling on reefs, which is driven by microorganisms within the coral holobiont. Occurrence of anaerobic processes in oxygenated bulk systems is potentially unexpected but not surprising. Numerous examples of the existence of micro-anaerobic sites exist in nature, from cyanobacterial and algal aggregates [76, 77] to marine snow particles [78] to sediments [79]. The governing principle in all of these systems is the same: biological oxygen consumption outpaces physical aeration via slow diffusion. Individual coral colonies are of a similar vein, especially given the mucus generating potential of the coral animal itself. This mucus acts not only to retard the diffusion of oxygen between bulk seawater but also as a carbon source, stimulating aerobic heterotrophy to actively draw down oxygen [40]. The thicker the mucus, the greater the potential for anaerobic processes to manifest even with bulk oxygen concentrations remaining too high to support denitrification within known oxygen constraints [80] (Fig. 6). As such, corals can host organisms that execute dissimilatory nitrate reduction, which can result in two pathways, each of which benefits the coral: (1) denitrification to N_2_O and N_2_ scrubs excess bio-available nitrogen that prevents macroalgal growth or (2) reduction to ammonium which permits more labile consumption of inorganic nitrogen for the coral and its symbionts [81].

The fact that ammonium oxidation rates were indistinguishable between coral-amended and seawater samples further supports the hypothesis that the mucus retards oxygenation and crafts a matrix less suitable for obligate aerobes. Indeed, the overall low rates of ammonium oxidation observed in the experiments were in line with expectations, whereby nitrification processes are inhibited by light through photo-stress [82] and from competition with ammonium assimilation by phototrophic organisms [83]. Low rates of ammonium oxidation in seawater around the corals may act to retard rates of nitrate reduction and denitrification in situ, as the supply of nitrate would be limited to flushing of the reef itself. However, nitrification by planktonic microbes may act to oxidize ammonium in seawater to nitrate, which is then supplied diffusionally to the coral associates. As such, the standing stock of nitrate concentrations can be maintained at the low (<0.4 µmol L^−1^) levels observed.

The dark experiments, compared to their diurnally varying light counterparts, also point to the prevalence of dissimilatory nitrate reduction metabolisms within the corals. The enhancement at low light of both nitrate reduction to nitrite and denitrifier production of N_2_O aligns with presumable periods of net respiration over photosynthesis among the coral-algae symbionts, conditions which would reduce oxygen and enable greater rates of anaerobic metabolisms. This is further confirmed by the observation that the amount of denitrification doubled for the dark experiments compared with an ~12/12 light/dark cycle in Jardines de la Reina. This agreement across the coral species sampled suggests anaerobic metabolisms are enhanced during the night-time only and relatively quiescent in daylight. This feature could be a result of oxygenic photosynthesis by the Symbiodinaceae algal symbionts reversibly inhibiting nitrate and nitrite reduction such that when oxygen production ceases due to insufficient light levels, nitrate and nitrite reduction become metabolically viable.

The benefits of dissimilatory nitrate and nitrite reduction within the coral holobiont are potentially substantial. Whereas these systems are severely limited by fixed nitrogen such that diazotrophs provide new nitrogen to the system, unchecked new nitrogen supplied by diazotrophy can lead to eutrophic conditions and the potential out-competition of zooxanthellae by benthic or planktonic algae. Greater nutrient availability can also lead to the unhindered growth of Symbiodinaceae itself and a shift from mutualistic to parasitic interactions with its coral host [84]. As such, a delicate balance between the nitrogen sources of fixation and sinks of denitrification and uptake needs to be maintained for a healthy reef ecosystem. Denitrifiers act to remove excess nitrogen that may otherwise stimulate algal production, and dissimilatory reducers of nitrate to ammonium may provide more labile nitrogen to zooxanthellae, especially during the night when assimilatory phototrophic nitrate and nitrite reduction may be limited. Furthermore, a healthy reef supports numerous fish, which can excrete large quantities of ammonium in close proximity of coral colonies, at rates similar to nitrogen fixation, which can stimulate growth of both *Porites* and *Acropora* corals [85]. Algae can rapidly assimilate these dynamic ammonium pulses [86] if not in competition with the holobiont for limited nitrogen resources.

Because of the double-edged nature of nitrogen nutrients, one can anticipate that a healthy reef would be one with a large diversity not just in organisms but also in metabolic pathways that act to maintain tight regulation of nutrient availability. Incredibly, the nitrate reduction rates observed here are on par with those observed in the anoxic marine oxygen minimum zones that are characterized by their hosting the denitrification metabolism [87, 88]. Indeed, evidence is emerging that denitrification is also important among analogous Red Sea corals [89, 90] The ability of natural coral-associated communities to reduce nitrate and denitrify is especially important to buffer against negative impacts in the Anthropocene, when increasing nitrogen supply can increase severity of coral disease [3, 4] and bleaching altogether [91].

The identification of the nitrate-reducing and denitrifying abilities of the coral holobiont allows a fuller picture of the nitrogen cycle associated with corals to be developed. Coral systems thrive despite the low nutrient availability because of the presence of diazotrophs in association with the corals, in the benthos, and among planktonic cells [16], but too much nitrogen fixation could cause macroalgae to flourish. Nitrogen fixation rates have been quantified among scleractinian corals to be highly species-specific, ranging from undetectable to ~2 nmol cm^−2^ d^−1^ in some [92, 93], to greater than 100 nmol cm^−2^ d^−1^ [16, 94] in others. The high variability in diazotrophy measurements indicates that the nitrate reduction and denitrification rates measured here may be significant contributors in the overall nitrogen balance for certain corals and at specific times. To that end, the ability of denitrifiers to remove excess nitrogen from the system can help maintain a healthy reef, otherwise imbalanced by the input of nitrogen fixation (or external nitrogen loading) and the output via nitrogen burial or advective transport. It can be hypothesized that without denitrifiers, the coral-Symbiodinaceae partnership could be outcompeted by other algae due to excess fixed nitrogen availability on the local scale, although importantly reef sediments too host denitrifying processes. However, denitrification is not the only dissimilatory nitrate consumption process. Dissimilatory reducers of nitrate to ammonium too were isolated from these corals, although we did not quantify rates of this process in situ. These organisms can potentially form a unique partnership with the coral and endosymbiotic algae too, in providing them a more readily labile reduced nitrogen source for assimilatory metabolisms. It has been shown for multiple scleractinian corals that ammonium is the preferred nitrogen substrate [95, 96]. Moreover, DNRA organisms may have the ability to detoxify nitrite, releasing N_2_O as a product. The results here point to the need to revisit the full complexity of the nitrogen metabolisms active among microbial coral associates, and the roles each has in maintaining a productive reef environment.

In summary, diazotrophy and nitrogen assimilation have understandably been the focus of many coral reef studies to date. However, as shown with this study, a diversity of metabolisms operating in concert with nitrogen fixation and nutrient uptake exists. The direct measurements of N_2_O from multiple ^15^N tracers are to our knowledge the first of their kind applied to tropical reef systems. The production of ^15^N_2_O from the ^15^NO_3_^–^ and ^15^NO_2_^–^ tracer experiments observed here provides strong evidence of fixed N loss from the system, from canonical and/or nitrifier denitrification, or detoxification. With nutrient pulses from anthropogenic effluent or ammonia excretion by fish [97], the importance of dissimilatory pathways could help maintain an overall balance [17, 98].

Coral health hinges on the dynamic relationship among members of the holobiont; microbial nitrogen cycling may play a central role in the maintenance of the seawater and boundary layer chemistry underlying the stability of the coral holobiont. The network of microbial nitrogen metabolisms is an especially important consideration in modern reefs, as anthropogenic nutrients, warmer temperatures, and decreased pH can alter the nitrogen speciation of the bulk water and the energetic yield of multiple metabolisms. These corals, like *Porites* and *Diploria*, form the basis of paleo-proxies used to understand previous climate and ocean states, and thus the isotopic fractionation involved with these dissimilatory metabolisms may need to be considered [99, 100]. Three lifestyles of anaerobic nitrogen bacteria were found here, nitrate-only reducers, denitrifiers, and DNRA organisms. The net effect this community has on overall nutrient cycling and biogeochemistry depends on the relative abundance and activities of these important microorganisms. Resolving this balance will rely on future measurements that monitor oxygen, preferably at the scale of the coral polyp itself, increase replication across coral species, and simultaneously measure rates of nitrogen fixation and denitrification.

## Supplementary information

Supplemental Material
