# Supplementary material for: Discovery and quantification of anaerobic nitrogen metabolisms among oxygenated tropical Cuban stony corals
Source: ISME J. 2020 Dec 20;15(4):1222–35. doi: 10.1038/s41396-020-00845-2 (PMC8115149; doi:10.1038/s41396-020-00845-2)
Supplement: Supplementary file 1 — Supplemental Material [file 41396_2020_845_MOESM1_ESM.pdf]

Supplementary Material for

Discovery and quantification of anaerobic nitrogen metabolisms among oxygenated tropical Cuban stony corals

Andrew R. Babbin, Tyler Tamasi, Diana Dumit, Laura Weber, María Victoria Iglesias Rodríguez, Sarah Schwartz, Maickel Armenteros, Scott D. Wankel, Amy Apprill

The supplementary materials include:

Supplementary Table 1. Physical characteristics of incubated coral fragments

Supplementary Table 2. Fixed nitrogen transformation rate data

Supplementary Table 3. Nitrous oxide production rate data

Supplementary Figure 1. Customized coral incubation vessels for ^{15}N tracer amendments

Supplementary Figure 2. *O. faveolata* example with ample mucus production

Supplementary Figure 3. Phenotypic nitrate and nitrite reduction assays results

Reef ID	Site #	Coral species	Surface area (cm^2)	Polyp count	Estimated dry weight (g)	Volume (cm^3)	Polyp density (polyps cm^{-2})
5B	1	*Porites astreoides*	7.1	191	0.19	3.9	2700
			8.3	212	0.22	3.4	2600
			6.3	182	0.17	2.6	2900
			5.2	142	0.13	1.6	2700
			14.3	331	0.22	2.8	2300
			11.2	301	0.16	4.1	2700
			9.3	247	0.21	2.9	2700
			7.8	191	0.20	2.7	2400
			4.5	123	0.11	3.2	2700
			6.8	162	0.11	1.1	2400
			7.1	187	0.19	1.7	2600
			8.8	236	0.19	2.0	2700
		mean ± sd	***8.1 ± 2.7***	***209 ± 61***	***0.18 ± 0.04***	***2.7 ± 0.9***	***2610 ± 160***
6B	2	*Porites porites*	25.3	703	0.26	5.9	2800
			12.5	351	0.19	2.5	2800
			8.1	285	1.35	1.4	3500
			18.4	567	0.67	3.0	3100
			21.0	563	0.24	3.6	2700
			18.8	524	0.20	4.3	2800
			13.9	376	0.22	3.8	2700
			8.6	248	0.15	2.1	2900
			7.9	218	0.21	1.5	2800
			19.3	589	0.10	4.2	3100
			24.2	611	0.21	4.5	2500
			21.6	595	0.27	7.8	2800
			16.6 ± 6.2	***469 ± 164***	***0.34 ± 0.35***	***3.7 ± 1.8***	***2860 ± 250***
10B	3	*Porites porites*	12.6	362	0.22	2.0	2900
			14.6	374	0.35	1.6	2600
			11.8	321	0.12	2.1	2700
			7.4	211	1.06	1.2	2900
			18.3	533	1.04	3.5	2900
			10.4	289	0.21	2.3	2800
			6.7	187	0.50	1.1	2800
			9.7	264	0.38	1.5	2700
			10.5	312	0.20	1.9	3000
			19.1	554	0.10	4.8	2900
			15.3	402	0.21	3.8	2600
			14.3	397	0.27	4.9	2800
			12.5 ± 3.9	***351 ± 113***	***0.39 ± 0.33***	***2.6 ± 1.3***	***2800 ± 120***
16C	4	*Orbicella faveolata*	4.9	22	0.20	3.1	450
			7.5	38	0.17	4.3	510
			3.6	21	0.14	7.2	590
			5.5	31	0.18	7.4	560
			5.6	36	0.18	4.7	640
			4.9	23	0.47	9.9	470
			5.0	24	0.19	4.2	480
			3.5	14	0.07	0.9	400
			5.1	18	0.11	7.8	360
			5.3	27	0.13	3.9	510
			6.4	38	0.15	6.1	590
			5.5	29	0.18	5.3	530
			5.2 ± 1.1	***27 ± 8***	***0.18 ± 0.10***	***5.4 ± 2.4***	***510 ± 80***

Supplementary Table 1. Characteristics of incubated coral fragments (continued on next page). The surface area was determined via wrapping the tissue-exposed portion of the fragment with aluminum foil and weighing the mold; polyps were directly counted; dry weight was estimated by weighing a portion of the sloughed tissue and mucus; skeletal volume was measured via water displacement. Polyps were not counted for the *D. labyrinthiformis* brain coral. The dry weight estimates were the least precise method due to the difficulty in homogenizing the slurry.

Reef ID	Site #	Species	Surface area (cm^2)	Polyp count	Estimated dry weight (g)	Volume (cm^3)	Polyp density (polyps cm^{-2})
18A	5	*Diploria labyrinthiformis*	3.4	ND	0.17	3.2	NA
			5.0	ND	0.15	4.9	NA
			5.8	ND	0.21	4.7	NA
			4.2	ND	0.12	9.8	NA
			5.1	ND	0.19	2.3	NA
			4.0	ND	0.04	3.1	NA
			3.3	ND	0.14	5.5	NA
			3.6	ND	0.06	3.8	NA
			2.9	ND	0.05	1.6	NA
			2.2	ND	0.07	1.9	NA
			8.0	ND	0.22	10.1	NA
			3.9	ND	0.17	2.5	NA
		mean ± sd	***4.3 ± 1.5***	—	***0.13 ± 0.06***	***4.5 ± 2.8***	—
15	6	*Acropora palmata*	33.4	287	0.25	7.2	860
			26.3	217	0.15	5.9	820
			35.8	317	0.21	8.3	890
			28.2	231	0.12	4.3	820
			21.9	212	0.11	3.9	970
			34.3	298	0.20	6.2	870
			37.0	306	0.15	5.1	830
			23.0	224	0.12	4.0	970
			22.9	226	0.09	3.6	990
			12.2	102	0.04	1.1	840
			17.4	147	0.10	5.7	840
			17.4	142	0.08	2.1	820
			25.8 ± 8.1	***226 ± 69***	***0.13 ± 0.06***	***4.8 ± 2.0***	***880 ± 60***
2B	7	*Orbicella faveolata*	5.1	33	0.12	1.5	650
			6.0	40	0.19	2.0	660
			11.7	81	0.22	5.7	690
			5.5	36	0.12	2.9	660
			4.8	33	0.06	2.2	690
			4.5	31	0.06	1.7	680
			9.3	58	0.07	5.9	620
			7.7	47	0.13	1.8	610
			6.4	41	1.26	1.2	640
			5.2	38	0.06	2.9	720
			2.9	21	0.03	1.4	730
			5.2	36	0.06	0.9	690
			6.2 ± 2.4	***41 ± 15***	***0.20 ± 0.34***	***2.5 ± 1.7***	***670 ± 40***

Supplementary Table 1 (continued). Physical characteristics of incubated coral fragments. The surface area was determined via wrapping the tissue-exposed portion of the fragment with aluminum foil and weighing the mold; polyps were directly counted; dry weight was estimated by weighing a portion of the sloughed tissue and mucus; volume was measured via water displacement. Polyps were not counted for the *D. labyrinthiformis* brain coral. The dry weight estimates were the least precise method due to the difficulty in homogenizing the slurry.

	NAR light		NAR dark		AMO light		AMO dark	
	Rep. 1	Rep. 2	Rep. 1	Rep. 2	Rep. 1	Rep. 2	Rep. 1	Rep. 2
P. astreoides	0.71	0.15	3.28	1.70	0.27	0.01	0.2	n.d.
P. porites **(Site 2)**	2.83	1.05	9.57	1.73	0.05	0.01	n.d.	n.d.
P. porites **(Site 3)**	2.35	1.73	3.74	1.36	n.d.	n.d.	n.d.	n.d.
O. faveolata **(Site 4)**	4.38	0.32	0.72	0.30	0.06	0.02	0.15	n.d.
O. faveolata **(Site 7)**	1.12	n.m.	1.32	n.m.	0.02	n.d.	0.02	n.d.
D. labyrinth.	1.72	1.14	2.42	2.32	n.d.	n.d.	0.03	n.d.
A. palmata	0.05	0.05	0.05	0.05	n.d.	n.d.	n.d.	n.d.

Supplementary Table 2. Fixed nitrogen transformation rate data. Rates (nmol-N cm^{-2} d^{-1}) of transformation for nitrate reduction (NAR) and ammonium oxidation (AMO) are shown for two replicates, for both diurnally varying (light) and 100% dark (dark) conditions. n.d., not detected; n.m., not measured.

	NO_3^- light		NO_3^- dark		NO_2^- light		NO_2^- dark		NH_4^+ light		NH_4^+ dark	
	Rep 1	Rep 2	Rep 1	Rep 2	Rep 1	Rep 2	Rep 1	Rep 2	Rep 1	Rep 2	Rep 1	Rep 2
P. astreoides	0.24	n.d.	0.59	0.41	0.34	0.06	0.51	0.47	n.d.	n.d.	n.d.	n.d.
***P. porites* (Site 2)**	0.45	0.13	0.54	n.d.	n.d.	n.d.	n.d.	n.d.	0.01	0.01	n.d.	n.d.
***P. porites* (Site 3)**	n.d.	n.d.	0.58	0.34	n.d.	n.d.	n.d.	n.d.	n.d.	n.d.	n.d.	n.d.
***O. faveolata* (Site 4)**	0.32	n.d.	0.05	0.05	1.00	n.d.	1.38	0.36	n.d.	n.d.	n.d.	n.d.
***O. faveolata* (Site 7)**	0.78	0.10	0.23	n.m.	1.74	0.38	1.06	0.62	0.02	n.d.	n.d.	n.d.
D. labyrinth.	3.10	1.06	6.55	1.09	2.87	2.71	10.62	5.74	0.06	0.02	0.13	0.03
A. palmata	0.16	n.d.	0.35	0.05	n.d.	n.d.	0.24	n.d.	n.d.	n.d.	n.d.	n.d.

Supplementary Table 3. Nitrous oxide production data. Rates (nmol-N cm^{-2} d^{-1}) of N_2O production from each nitrogen pool (NO_3^-, NO_2^-, and NH_4^+) are shown for two replicates, for both diurnally varying (light) and 100% dark (dark) conditions. n.d., not detected; n.m., not measured.

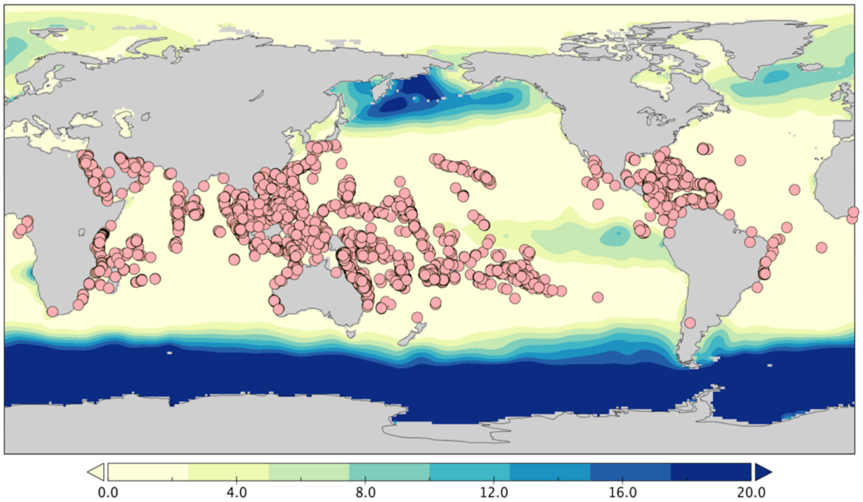

Supplementary Figure 1. Coral reefs co-locate where surface nitrate levels are low. Shown are the locations of modern coral reefs (circles) from reefdata.org overlain on a surface map of nitrate concentrations from World Ocean Atlas 2018.

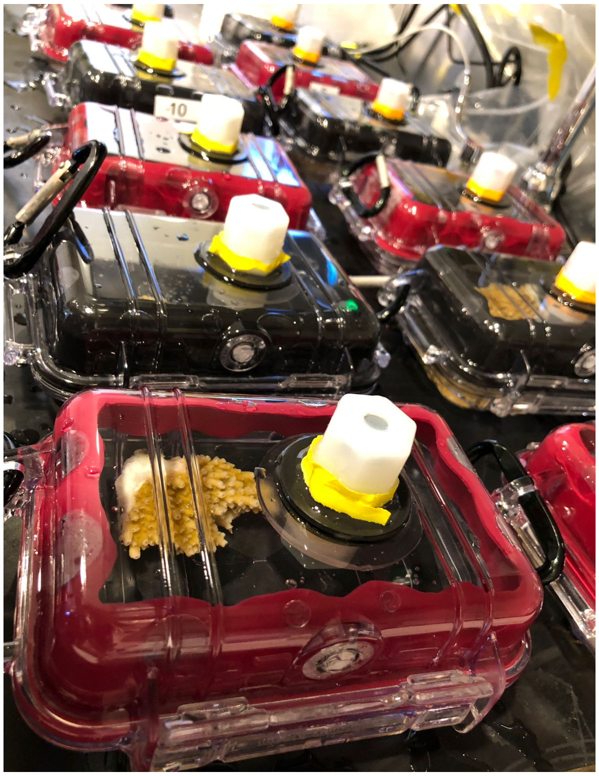

Supplementary Figure 2. Customized coral incubation vessels for ^{15}N tracer amendments. The incubation vessels were constructed from water-tight polycarbonate chambers that were customized with a through-wall bulkhead fitting. Gas-tightness of this connection was ensured with a Viton rubber gasket secured with a polycarbonate washer. The bulkhead fitting contained a chlorobuytl rubber septum (Labco, UK). The chambers were filled completed with reef water and a piece of coral placed in each chamber. Afterward, a mixture of nutrient amendments were injected through the septum port. 18 replicate chambers were used for an experiment, consisting of 6 for each ^{15}N tracer: 2 light, 2 dark, and 2 controls without corals.

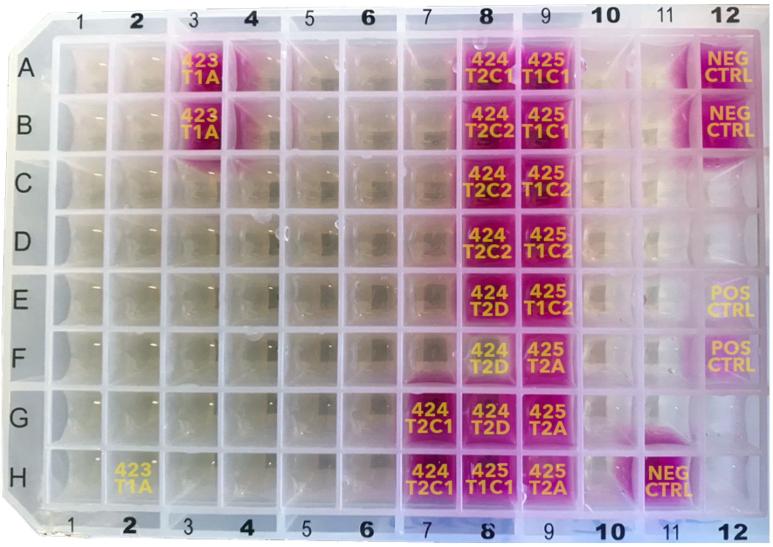

Supplementary Figure 3. Phenotypic reduction assays results. Deep 96-well plates were amended with 50 µmol L^{-1} nitrate or nitrite and allowed to incubate anaerobically. Each culture was amended into triplicate wells. Nitrite production (from nitrate) or consumption (from nitrite) were observed using the colorimetric Griess assay. This photograph is of the nitrite assay at 144 hours, although the same results are observed for all time points. The negative control was unamended with bacteria whereas the positive control was amended with *Pseudomonas aureofaciens*, a denitrifier able to reduce nitrite slowly. The annotations are of the following: 423-T1A (*Pseudoalteromonas sp.* from *P. astreoides*), 424-T2C and D (*Pseudoalteromonas sp.* from *M. cavernosa*), 425-T1C1 (*Pseudoalteromonas sp.* from *D. labyrinthiformis*), 425-T1C2 and T2A (*Psychrobacter sp.* from *D. labyrinthiformis*). The lack of color observed in H2 and F8 is attributed to cross-contamination in the inocula of those individual wells with a nitrite-reducing organism.
